# “Effect of agroecological and conventional farming systems on the metabolomic profile of yellow and red maize assessed by ^1^H NMR”

**DOI:** 10.1016/j.fochx.2026.103508

**Published:** 2026-01-07

**Authors:** Gustavo G. Medina-Mendoza, Elvia Becerra-Martínez, Gerardo Noriega-Altamirano, José Javier Castro-Arellano, Oscar Camacho-Nieto, Diego Hidalgo-Martínez, Yair Cruz-Narváez

**Affiliations:** a Instituto Politécnico Nacional-ESIQIE-UPALM, Laboratorio de Posgrado e Investigación en Operaciones Unitarias. Edificio 7, 1.er Piso, Sección A, Av. Luis Enrique Erro S/n, Unidad Profesional Adolfo López Mateos, Zacatenco, C. P. 07738, Delegación Gustavo A. Madero, Ciudad de México, Mexico; b Centro de Nanociencias y Micro y Nanotecnologías, Instituto Politécnico Nacional, Av. Luis Enrique Erro S/N, Unidad Profesional Adolfo López Mateos, Zacatenco, Delegación Gustavo A. Madero, Ciudad de México 07738, Mexico; c Universidad Autónoma Chapingo, Academia de Meteorología, Área de Agronomía. km 38.5 Carretera México-Texcoco. C. P. 56230. Chapingo, Texcoco, Estado de México, Mexico; d Centro de Innovación y Desarrollo Tecnológico en Cómputo del Instituto Politécnico Nacional, Av. Juan de Dios Bátiz s/n, Nueva Industrial Vallejo, Gustavo A. Madero, 07700 Ciudad de México, D.F., Mexico; e Department of Biology, Healthcare and the Environment, Faculty of Pharmacy and Food Sciences, University of Barcelona, 08028 Barcelona, Spain. Maria de Maeztu Unit of Excellence, Institute of Nutrition and Food Safety, University of Barcelona (INSA-UB), 08921 Santa Coloma de Gramenet, Spain

**Keywords:** Maize, Agroecological, ^1^H NMR, Metabolomic profile, Multivariate analysis

## Abstract

The growing demand for nutritionally high-quality foods produced under sustainable schemes has driven evaluation of how agricultural systems influence maize chemical composition. Conventional agriculture relies heavily on chemical inputs, affecting environmental sustainability and grain quality, whereas agroecological systems represent a lower-impact alternative; however, their effects on the metabolome of native maize remain insufficiently understood. This study compared the metabolomic profiles of two native Mexican maize varieties, Zamorano yellow (MZ) and Chalqueño red (PR), cultivated under agroecological and conventional management. ^1^H NMR spectroscopy with PCA and OPLS-DA analyses was used to identify differential metabolites. Metabolic discrimination between management systems was observed. Agroecological management affected tricarboxylic acid cycle, pyruvate, and glyoxylate metabolism in MZ, and amino acid biosynthesis and sugar metabolism in PR, increasing the abundance of 17 metabolites in MZ and 15 in PR. These findings highlight the potential of agroecological management to enhance maize nutritional quality within sustainable production systems.

## Highlights

• Agroecological management reprograms maize metabolism within each variety.
• Zamorano maize accumulates organic acids, sugars, and GABA under agroecological management.
• Red maize shows increased amino acids and phenolic precursors under agroecological management.
• OPLS-DA discriminates agroecological and conventional systems in a variety-dependent manner.
• Agroecological management enhances metabolic traits associated with grain nutritional quality.

## Graphical abstract

**Figure:**
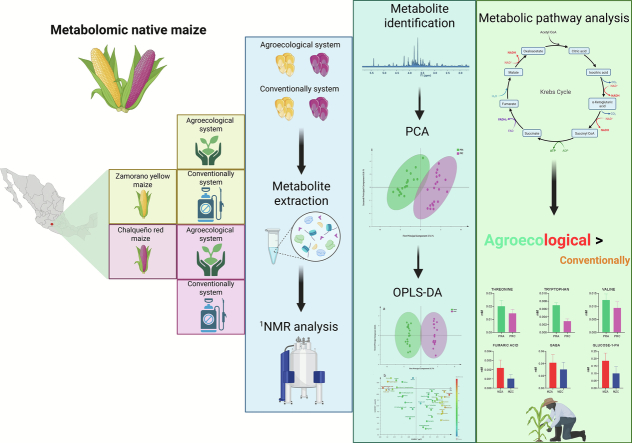

## 1 Introduction

Maize (*Zea mays* L.) is one of the most widely cultivated and consumed cereals worldwide, together with wheat and rice, playing a fundamental role in global food security (Zhang et al., 2020). Its nutritional value and versatility make it an essential food in both developed countries and developing regions. In Mexico, maize has even greater relevance, not only as a staple food but also as a cultural, economic, and biotechnological resource.

Given its nutritional importance, maize has been positioned as a strategic crop. However, its chemical composition can vary significantly due to factors such as genetic variety, growing environment, and particularly the type of agricultural management applied. Conventional management is based on the intensive use of synthetic fertilizers, pesticides, and machinery, which has allowed increased productivity. Nevertheless, it has also generated negative environmental impacts, such as soil degradation and contamination of water bodies(Song et al., 2023).

Conventional agriculture has been the dominant production model since the Second World War. However, this system is characterized by a high dependence on external inputs, particularly synthetic fertilizers and pesticides, which has raised concerns regarding its long-term environmental sustainability(Gliessman, 2016). Prolonged implementation of conventional agricultural systems has been associated with soil degradation processes, including reductions in fertility and soil quality, representing a significant challenge for crop sustainability (Martínez-Aguilar et al., 2020). n this context, the adoption of soil restoration and sustainable management practices has gained importance, given that soil constitutes a non-renewable resource and an essential component for food production and food security (Garay Peralta et al., 2022).

In contrast, the agroecological model promotes sustainable agricultural practices that respect ecological cycles and conserve biodiversity(Altieri & Nicholls, 2000; Pretty, 2018). Several studies have shown that crops grown under agroecological systems may present higher concentrations of bioactive compounds, such as polyphenols, flavonoids, and carotenoids, resulting in greater nutritional value(Giri & Pokhrel, 2022).

These systems also contribute to improving soil structure and reducing greenhouse gas emissions, positioning them as a viable alternative in the context of climate change (Gattinger et al., 2012).

Agroecological management also promotes the synthesis of key metabolites involved in crop development and resistance to abiotic and biotic stress. Primary metabolites, such as sugars, amino acids, and lipids, are essential for basic cellular functions, whereas secondary metabolites, including phenolics, alkaloids, and flavonoids, play an important role in plant defense under adverse conditions (Garcia-Baeza et al., 2023). In addition, many of these compounds exhibit beneficial effects on human health, including antioxidant, anti-inflammatory, and hepatoprotective properties (Zhang et al., 2020).

Over the past decade, advances in omics sciences particularly genomics, transcriptomics, proteomics, and metabolomics have enabled a more detailed characterization of maize and its varieties (Medeiros et al., 2021). Among these tools, proton nuclear magnetic resonance spectroscopy (^1^H NMR) has become an important technique for metabolomic analysis, as it allows the detection and quantification of metabolites with minimal sample processing, in a non-destructive manner and with high reproducibility (Gupta & Gaur, 2024). This technique has been successfully applied to agricultural crops to analyze the impact of genetic, environmental, and management factors (Emwas et al., 2019), and has demonstrated its usefulness in the food and pharmaceutical industries (Montoya-García et al., 2023).

Michoacán exhibits a rich genetic diversity, hosting 27 of the 64 native maize races, including Cónico, Chalqueño, Elotes Cónicos, Elotes Occidentales, Tabloncillo, Cacahuazintle, Palomero, Toluqueño, Ancho, Mushito, Pepitilla, among others. In this context, the present study conducted the metabolomic characterization of two maize varieties native to Mexico: Zamorano yellow maize and red maize. Maize represents a relevant source of micronutrients, including B-complex vitamins, as well as vitamins A and E, contributing to its importance in human nutrition (Elisa et al., 2022), Due to its nutritional value and wide diversity of uses, maize plays a fundamental role in traditional Mexican gastronomy (Pérez Ruiz et al., 2024). In particular, pigmented maize varieties, displaying colors ranging from blue and black to red, purple, and cherry, have been widely recognized for their high content of bioactive compounds, such as anthocyanins, carotenoids, bioactive peptides, and phenolic acids, which are associated with nutraceutical properties (Reyes-Pavón et al., 2024) and potential applications in the nutrition, medical, and food industries (Domínguez-Hernández et al., 2025).

The objective of this study was to compare the metabolomic profiles of two native Mexican maize varieties cultivated under agroecological and conventional management systems in two regions of the state of Michoacán, Mexico. Zamorano yellow maize was collected in Contepec (19.87° N, 100.29° W), while Chalqueño red maize was collected in Zacapu (19.82° N, 101.79° W). The analysis was carried out using proton nuclear magnetic resonance spectroscopy (^1^H NMR), allowing the generation of a detailed profile of their chemical and metabolomic composition, and evaluating how management type influences the accumulation of compounds with nutritional and functional value, contributing to the revalorization of these varieties for diverse applications.

## 2 Materials and methods

### 2.1 Cultivation areas and varieties

Two maize varieties were evaluated: Zamorano yellow maize (MZ), collected from the locality of Contepec, Michoacán (Fig. S1), which was grown during the spring–summer cropping cycle at an elevation of 2497 m above sea level. The second variety was red maize (PR), locally known as “red pozole maize,” collected from the locality of Zacapu, Michoacán (Fig. S2), also cultivated during the spring–summer cycle at an elevation of 1981 m above sea level.

According to the official edaphological cartography of the *Instituto Nacional de Estadística y Geografía (INEGI)*, the municipality of Zacapu, Michoacán, is characterized by the presence of Histosols (INEGI, 2010b), particularly associated with wetland and ciénega environments, whereas Vertisols are reported as one of the main soil groups in the municipality of Contepec, (INEGI, 2010a) Michoacán. These edaphic conditions reflect contrasting geomorphological and hydrological environments, which were considered as part of the environmental context for the interpretation of the agronomic and metabolomic results of the present study.

### 2.2 Types of agricultural management

*Conventional Management:* Conventional management was carried out according to the protocol recommended by the National Institute for Forestry, Agricultural and Livestock Research (INIFAP), which specifies the dosage of agrochemicals and pesticides recommended in the technical guidelines for maize production within the Agricultural Technical Agenda of Michoacán (INIFAP, 2015).

Soil fertilization was performed through irrigation, supplying 90–45–30 (kg/ha) of nitrogen, phosphorus, and potassium (N–P–K), respectively, in two applications. The first application was conducted at sowing, using urea as the nitrogen source, mixed with diammonium phosphate (DAP). The second application was carried out when maize plants reached the eighth to tenth ligulated leaf stage, followed by the application of pesticides as recommended in the technical guide.

*Agroecological* Management: The research group established the following management scheme, based on the application of three in-house developed biological products, consisting of a microbial consortium, natural zeolite, a biostimulant, and a foliar fertilizer. Applications were performed through surface irrigation and foliar spraying twice per week. Prior land preparation, the incorporation of enriched compost, and weed management were carried out manually, without the use of synthetic chemical products.

The key stages of this agroecological technology included: (1) Restoration tillage. (2) Precision sowing and population density management to optimize solar radiation capture. (3) Rational fertilization based on soil fertility diagnosis. (4) Soil remineralization to correct pH, cation exchange capacity (CEC), and degradation processes. (5) Restoration of soil organic matter and biological activity. (6) Complementary nutrition through foliar applications. (7) Biological pest and disease management through the application of a microbial consortium inoculated into the soil and applied foliar.

### 2.3 Sampling methodology

Two maize varieties, Zamorano yellow and red (pozole maize), cultivated under two agricultural management systems conventional and agroecological were collected. Based on this combination, four experimental groups were established: agroecological Zamorano yellow maize (MZA), conventional Zamorano yellow maize (MZC), agroecological red maize (PRA), and conventional red maize (PRC). For each group, three independent cultivation blocks were established (Fig. S3), resulting in a total of 21 ears per experimental group.

To ensure biological representativeness and reduce bias associated with intra-plot variability, stratified random sampling by ear size was applied. Within each cultivation block, five linear meters per row were selected, from which all ears from plants that had reached physiological maturity at the time of harvest were collected. The ears harvested from each row were arranged in descending order according to size and overall morphological development (Fig. S4). Based on this ordering, three representative categories were identified: the smallest ear, the medium-sized ear (corresponding to the population median), and the largest ear. This procedure allowed capturing the natural variability of the crop and ensured that the selected sample adequately reflected the morphological heterogeneity within each experimental group.

From this set, seven representative ears per group were selected, integrating samples from different rows and cultivation blocks. These were considered biological replicates for subsequent analyses.

The selected ears were individually stored in polyethylene bags, properly labeled with the corresponding row and replicate number (e.g., Row 1, Replicate 1; R1, Rep1), and transported to the laboratory for processing and metabolomic analysis.

### 2.4 Planting density

For Zamorano yellow maize, row spacing was set at 0.80 m with 0.18 m between plants, resulting in 555 plants per 100-m row and a density of 69,444 plants per hectare.

For Chalqueño red maize, row spacing was 0.80 m with 0.33 m between plants, yielding 333 plants per 100-m row and a density of 41,625 plants per hectare.

### 2.5 Standards and reagents

The analyses were performed using the following standards and reagents: deuterium oxide (D₂O, 99.9% D) obtained from Cambridge Isotope Laboratories; sodium azide (NaN₃); trimethylsilylpropanoic acid (TSP-d₄, 98%); potassium phosphate (KH₂PO₄); and ethylenediaminetetraacetic acid (EDTA), all sourced from Sigma Aldrich.

### 2.6 ^1^H NMR analysis

Maize samples were ground in a Bogner electric mill to obtain a fine, homogeneous powder, then lyophilized under 1 Pa vacuum pressure for 24 h. at −60 °C. Fifty milligrams of sample were mixed with 725 μL of D₂O and 75 μL of a solution containing 90 mM KH₂PO₄, 2 mM NaN₃, 2 mM TSP-d₄, and 10 mM EDTA in D₂O (Becerra-Martínez et al., 2017) Samples were homogenized by sonication for 20 min at 27 °C, centrifuged for 40 min at 10,000 rpm, filtered through PES membranes (13 mm diameter, 0.22 μm pore size), and 600 μL of the supernatant was transferred into 5 mm NMR tubes.

### 2.7 ^1^H NRM equipement and analysis conditions

Samples were analyzed using a Bruker 750 MHz spectrometer (Bruker BioSpin, Rheinstetten, Germany) equipped with a 5 mm TXI cryoprobe. Aqueous maize extracts were measured at 298.1 ± 0.1 K without spinning, using 512 scans. Acquisition parameters were: FID size = 64 K; spectral width = 9.99 ppm; receiver gain = 167; acquisition time = 2.18 s; relaxation delay = 5 s; and FID resolution = 0.23 Hz. Data acquisition employed the *noesypr1d* pulse sequence with water suppression by irradiation at the water frequency during recycle and mixing times. (Villa-Ruano et al., 2020).

### 2.8 Metabolite identification

^1^H NMR spectra were processed with Chenomx NMR Suite version 10.0 (Chenomx, Edmonton, Canada), baseline-corrected, phase-adjusted, and calibrated using the internal standard (TSP) signal. The pH value was entered within a specific range (pH 4–9). Primary metabolites from the two maize varieties were identified and quantified, and the concentration data were subjected to multivariate analysis using SIMCA (v13.0.3, Umetrics, Umea, Sweden).

### 2.9 Multivariate statistical analysis

Data were log-normalized and UV-scaled. Principal component analysis (PCA) was performed to assess general variation patterns, identify clustering, and detect potential outliers. Orthogonal projections to latent structures discriminant analysis (OPLS-DA) was used to separate groups according to farming system (agroecological vs. conventional). Model validation was performed using permutation tests (*n* = 200) and assessed with R^2^X(cum), R^2^Y(cum), and Q^2^(cum) parameters. (Liu et al., 2023). Differential metabolites were identified using Variable Importance in Projection (VIP) scores ≥1, which were then used for metabolic pathway analysis. Tukey's test was applied to identify statistically significant differences (*p* < 0.05) between metabolite concentrations under both management systems.

## 3 Results and discussion

### 3.1 Averange yield

The average yield under conventional management was 7.6 t ha^−1^ for the Zamorano yellow maize (MZ) variety and 3.65 t ha^−1^ for the Chalqueño red maize (PR). Under agroecological management, the yield of Zamorano yellow maize remained practically unchanged (7.7 t ha^−1^; +0.1 t ha^−1^ compared with conventional), whereas Chalqueño red maize exhibited a marked increase, reaching 12.8 t ha^−1^ approximately 9.15 t ha^−1^ higher than under conventional management. Thus, a significant yield improvement was observed only in the Chalqueño red maize variety cultivated under the agroecological system.

### 3.2 Metabolic profile of maize varieties

The ^1^H NMR spectra at 750 MHz of aqueous extracts from Zamorano and Chalqueño red maize varieties, each cultivated under agroecological and conventional management, are shown in Fig. S5. A total of 49 metabolites were identified based on chemical shifts from 2D spectra, literature reports, and the Human Metabolome Database (http://www.hmdb.ca). The corresponding assignments are presented in Tabla S1.

Fig. S6a displays the representative ^1^H NMR spectrum of maize extracts over the entire spectral range (0.5–9.5 ppm), before individual signal assignment. This global spectrum provides an overview of the chemical diversity present in the samples, with the most intense resonances between 0.8 and 4.5 ppm corresponding mainly to aliphatic hydrogens from amino acids, organic acids, and carbohydrates. The smaller peaks in the aromatic region (6.0–9.0 ppm) correspond to phenolic compounds and nucleosides. This overall profile was used as a reference for phase and baseline correction, spectral alignment, and calibration using the internal standard (TSP-d₄ at 0 ppm).

Figs. S6b-d show expanded spectral regions highlighting the main metabolite classes detected. In the aliphatic region (0.85–2.2 ppm), signals correspond primarily to branched-chain and small amino acids such as valine, leucine, isoleucine, alanine, and proline, together with osmolytes including γ-aminobutyric acid (GABA).

The central region (2.3–5.5 ppm) is dominated by carbohydrates such as glucose, fructose, sucrose, maltose, and myo-inositol, which are characteristic of maize endosperm metabolism. Finally, the aromatic region (5.8–9.2 ppm) includes compounds such as phenylalanine, tyrosine, tryptophan, formic acid, fumaric acid, trigonelline, and adenosine, which represent the connection between primary and secondary metabolism.

Overall, sugars, amino acids, organic acids, and nucleosides were the predominant metabolite groups in both maize varieties. These results are consistent with previous ^1^H NMR metabolomic studies in cereals(Sobolev et al., 2024) and provide a robust chemical basis for subsequent multivariate analyses. A complete list of identified metabolites, their chemical shifts, and multiplicities is provided in Table S1 of the Supplementary Material.

### 3.3 Multivariate data analysis and metabolic classification by management type

To evaluate differences in metabolomic profiles associated with agricultural management type, a multivariate approach based on principal component analysis (PCA) and orthogonal partial least squares discriminant analysis (OPLS-DA) was applied, considering each maize variety independently.

For the Zamorano yellow maize (MZ), a three-component PCA explained 45.5% of the total variance of the dataset, with the first principal component (PC1) accounting for 26.6% and the second principal component (PC2) for 18.9%. The score plot showed a clear separation between agroecological (MZA) and conventional (MZC) management systems (Fig. S7), indicating that management type represents a relevant source of metabolic variation in this variety.

**Fig. 1:**
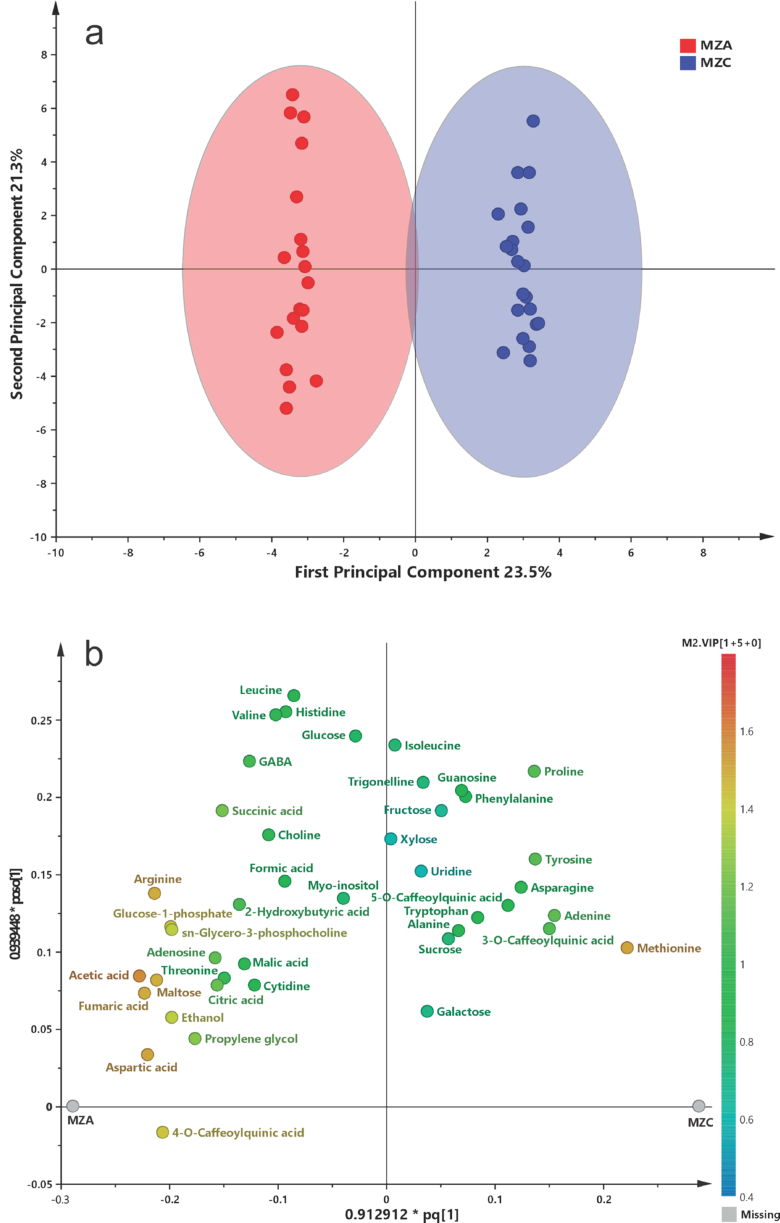
**(a)** OPLS-DA score plots generated from concentration values obtained by ^1^H NMR (750 MHz) of Zamorano yellow maize under agroecological (MZA, red) and conventional (MZC, blue) management. **(b)** Loading plots illustrating the distribution of the most significant metabolites. (For interpretation of the references to colour in this figure legend, the reader is referred to the web version of this article.)

To maximize group discrimination and identify the metabolites responsible for this separation, an OPLS-DA model was constructed using two predictive and three orthogonal components. The model showed values of R^2^X(cum) = 0.550, R^2^Y(cum) = 0.931, and Q^2^(cum) = 0.701 (Fig. 1a), indicating an adequate model fit and good predictive ability. Model validity was confirmed through permutation testing (*n* = 200). Based on loading analysis and variable importance in projection (VIP ≥ 1) values, 20 metabolites with significant differences between management systems were identified (Table S2, Fig. S8).

**Fig. 2:**
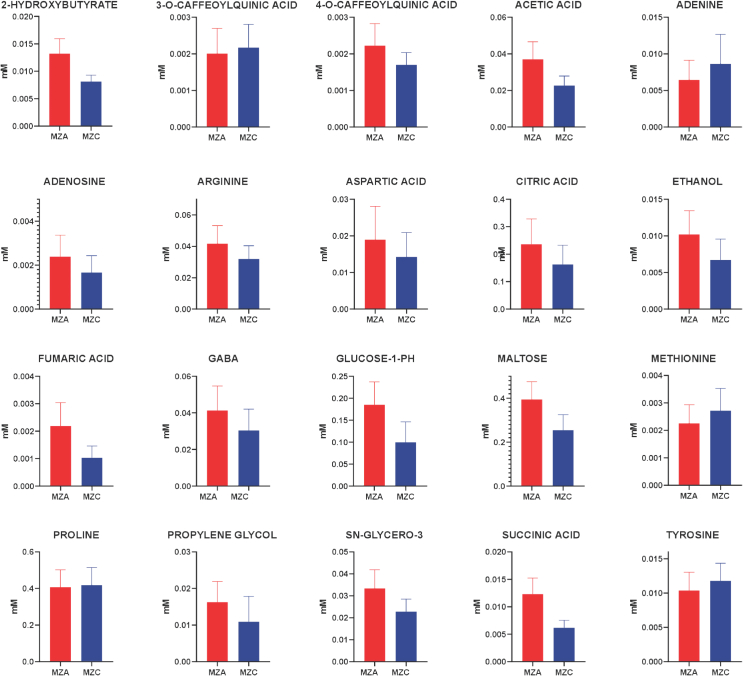
Relative concentration of 20 significant metabolites (VIP score ≥ 1) in Zamorano yellow maize under agroecological (MZA, red) and conventional (MZC, blue) management. Relative concentration values are expressed in mM. (For interpretation of the references to colour in this figure legend, the reader is referred to the web version of this article.)

Zamorano yellow maize grown under agroecological management was characterized by higher relative concentrations of amino acids, organic acids, and carbohydrates, including 2-hydroxybutyrate, aspartate, glutamate, GABA, citrate, succinate, fumarate, maltose, and glucose-1-phosphate, among others. These metabolites are closely associated with central carbon and nitrogen metabolism, particularly the tricarboxylic acid (TCA) cycle, pyruvate metabolism, and pathways related to amino acid biosynthesis. Collectively, these changes suggest a reorganization of primary metabolism induced by agroecological management, reflected in a redistribution of carbon and nitrogen fluxes at the metabolic level, rather than a direct measurement of nitrogen use efficiency. The relative concentrations of the most significant metabolites are shown in Fig. 2.

For the red maize variety (PR), a three-component PCA explained 46.2% of the total variance, with PC1 accounting for 37.6% and PC2 for 8.6%. Similar to the observations in MZ, PCA analysis revealed a clear separation between samples under agroecological (PRA) and conventional (PRC) management (Fig. S9), confirming the effect of the management system on the metabolomic profile of this variety.

**Fig. 3:**
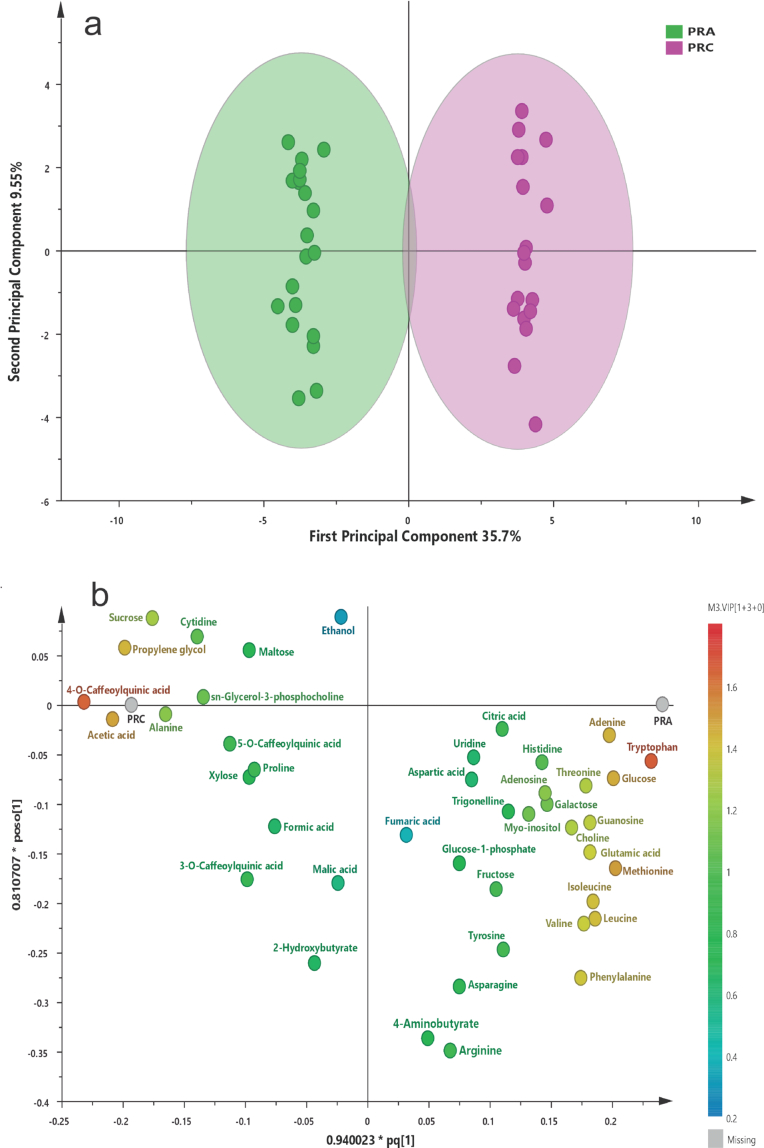
**(a)** OPLS-DA score plots generated from concentration values obtained by ^1^H NMR (750 MHz) of Chalqueño red maize under agroecological (PRA, green) and conventional (PRC, pink) management. **(b)** Loading plots illustrating the distribution of the most significant metabolites. (For interpretation of the references to colour in this figure legend, the reader is referred to the web version of this article.)

The OPLS-DA model constructed for red maize included two predictive and three orthogonal components and showed values of R^2^X(cum) = 0.545, R^2^Y(cum) = 0.992, and Q^2^(cum) = 0.954 (Fig. 3a), indicating a highly robust model with excellent predictive performance, validated by permutation testing (*n* = 200). Based on VIP values (≥ 1), 18 metabolites with significant differences between management systems were identified (Table S3, Fig. S10).

**Fig. 4:**
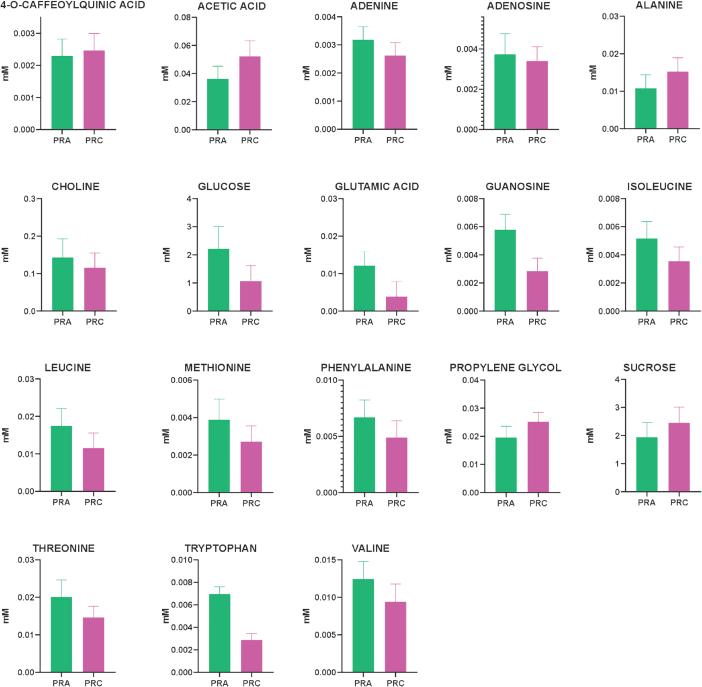
Relative concentration of the 18 significant metabolites (VIP score ≥ 1) in Chalqueño red maize under agroecological (PRA, green) and conventional (PRC, pink) management. Concentration values are expressed in Mm. (For interpretation of the references to colour in this figure legend, the reader is referred to the web version of this article.)

Red maize under agroecological management exhibited higher relative concentrations of nucleosides, amino acids, and sugars, including adenine, adenosine, choline, glucose, glutamate, and guanosine, as well as essential amino acids such as isoleucine, leucine, methionine, phenylalanine, threonine, tryptophan, and valine. These metabolites are associated with amino acid biosynthesis pathways, energy metabolism, and regulation of nitrogen metabolism, suggesting a differentiated metabolic response linked to agroecological management, supported by the observed metabolomic profile rather than by direct physiological measurements. The relative concentrations of the most relevant metabolites are presented in Fig. 4.

### 3.4 Metabolic pathway analysis of Zamorano yellow and Chalqueño red maize under conventional and agroecological management

The identification of changes in the metabolic profiles of both maize varieties was carried out through metabolic pathway analysis using MetaboAnalyst 6.0, integrating statistical and topological tools to determine the degree of metabolic perturbation based on quantified metabolite levels and their position within the metabolic network.

The selection of relevant metabolic pathways was based on two main criteria: statistical significance (FDR < 0.05) and topological impact (impact >0.1), calculated using the *Relative Betweenness Centrality* algorithm and the KEGG database, following methodologies previously standardized in metabolomic studies (Xia et al., 2015) (Chong et al., 2019).

In Zamorano yellow maize, significant differences were identified in 11 metabolic pathways (Fig. S11, Table S4). In this study, pathways with a topological impact >0.3 and an FDR < 0.05 were considered relevant, prioritizing those that combine statistical significance with functional centrality within the metabolic network. Based on these criteria, three major pathways stood out: alanine, aspartate, and glutamate metabolism (Impact = 0.77698; FDR = 4.93E−05), pyruvate metabolism (Impact = 0.38783; FDR = 4.93E−05), and starch and sucrose metabolism (Impact = 0.59548; FDR = 0.00445).

**Fig. 5:**
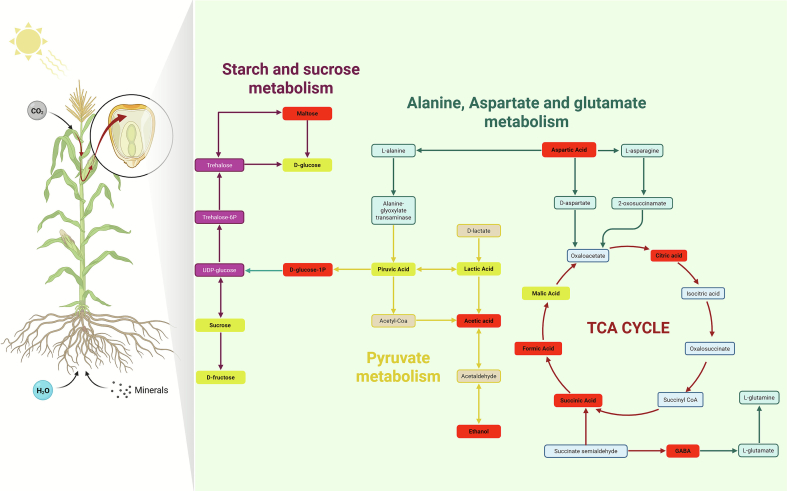
Metabolic pathways affected in Zamorano yellow maize (MZ), including starch and sucrose metabolism, pyruvate metabolism, and alanine, aspartate, and glutamate metabolism, highlighting metabolites with the highest discriminant value (VIP score ≥ 1, red). (For interpretation of the references to colour in this figure legend, the reader is referred to the web version of this article.)

These pathways are highly interconnected, forming key nodes in central carbon and nitrogen metabolism (Fig. 5). Starch and sucrose metabolism generates precursors such as d-glucose, d-glucose-1-phosphate, and d-fructose, which feed into glycolysis and subsequently produce pyruvate. Pyruvate acts as a metabolic junction linking glycolysis, the tricarboxylic acid (TCA) cycle, and acetyl-CoA production, thereby regulating energy balance and the synthesis of organic acids, ethanol, and lactate(Thalmann & Santelia, 2017; Zeeman et al., 2010).

Alanine, aspartate, and glutamate metabolism directly connects the TCA cycle with the biosynthesis and degradation of non-essential amino acids, integrating carbon flux with nitrogen assimilation and transport. This pathway plays a central role in stress responses and in the synthesis of essential amino acids (Forde & Lea, 2007; Liao et al., 2022). Metabolites identified as representative through OPLS-DA analysis (VIP ≥ 1), including maltose, d-glucose, d-glucose-1-phosphate, aspartic acid, citric acid, formic acid, succinic acid, acetic acid, and GABA, represent strategic control and connection points between pathways, reinforcing their role in discriminating between the evaluated management systems (Alam et al., 2025; Nunes-Nesi et al., 2011).

The interrelationship among these pathways indicates that maize central metabolism does not operate in isolation but rather as an integrated network linking cellular respiration, energy generation, primary metabolite synthesis, and the production of secondary metabolites with nutraceutical relevance. This metabolic organization, observed more prominently under agroecological management, is consistent with a coordinated redistribution of carbon and nitrogen fluxes at the metabolic level. However, since nitrogen use efficiency was not directly evaluated in this study, these observations should be interpreted as indirect evidence based on metabolomic profiles rather than as direct physiological measurements (; Barański et al., 2014; Guo et al., 2021).

**Fig. 6:**
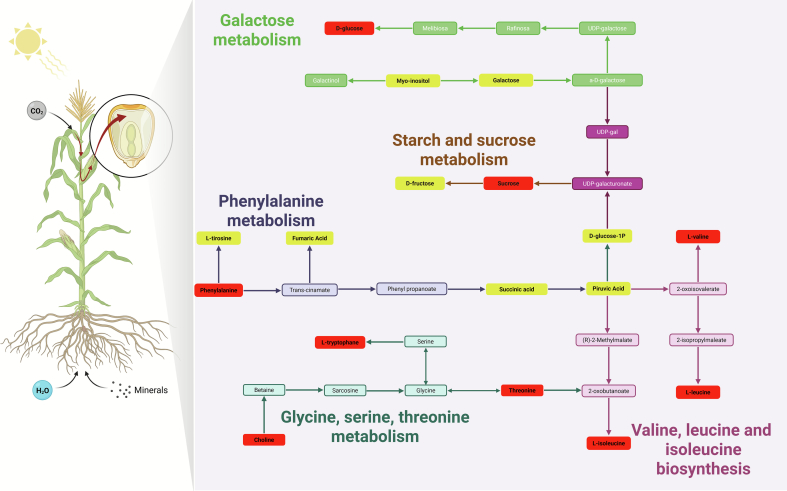
Metabolic pathways affected in Chalqueño red maize (PR), including galactose metabolism, starch and sucrose metabolism, phenylalanine metabolism, glycine, serine, and threonine metabolism, and the biosynthesis of valine, leucine, and isoleucine, highlighting metabolites with the highest discriminant value (VIP score ≥ 1, red). (For interpretation of the references to colour in this figure legend, the reader is referred to the web version of this article.)

According to the pathway selection criteria, the red maize variety (PR) exhibited significant differences in 10 metabolic pathways (Fig. S12, Table S5). Three major pathways were identified (Fig. 6): starch and sucrose metabolism (Impact = 0.59548; FDR = 0.002152), phenylalanine metabolism (Impact = 0.42308; FDR = 0.0073737), and galactose metabolism (Impact = 0.34927; FDR = 0.0083761).

Within galactose metabolism, the conversion of d-glucose to galactinol was identified through intermediates such as melibiose, raffinose, α-D-galactose, and UDP-galactose, converging with starch and sucrose metabolism. The latter generates precursors such as d-glucose-1-phosphate, d-fructose, and sucrose, which feed pyruvate synthesis and connect with biosynthetic and energy-generating pathways. Starch and sucrose metabolism plays a critical role in carbohydrate assimilation and storage, regulating energy availability and adaptive responses to changing environmental conditions (Tzin & Galili, 2010).

Additionally, glycine, serine, and threonine metabolism integrates amino acids and derivatives such as choline, betaine, sarcosine, glycine, serine, and threonine, linking them to branched-chain amino acid biosynthesis and regulating nitrogen compound formation and carbon flux (Ros et al., 2014).

These highly relevant central metabolic pathways exhibited convergence points with other biologically important routes, such as the biosynthesis of valine, leucine, and isoleucine—branched-chain amino acids essential for growth and stress responses (Binder, 2010) and with glycine, serine, and threonine metabolism, which plays a key role in nitrogen compound formation and carbon flux regulation.

Metabolites highlighted in red in the metabolic network (Fig. 6) correspond to representative compounds with VIP values ≥1 in the OPLS-DA analysis, indicating their relevance in differentiating between management systems. These include d-glucose, sucrose, L-valine, L-leucine, L-isoleucine, phenylalanine, choline, and L-tryptophan, which represent key nodes in carbon and nitrogen flux. Metabolites shown in yellow were detected but not statistically significant, while green and purple metabolites represent non-differential intermediates that help visualize pathway interconnections.

Overall, these pathways and metabolites reflect greater carbohydrate use efficiency and activation of secondary pathways associated with bioactive compound synthesis under agroecological management, which may contribute to improved adaptation of red maize to low agrochemical input conditions and enhanced nutritional and functional grain quality (Knapp et al., 2023; Ponisio et al., 2015).

In total, 20 discriminant metabolites were identified in Zamorano yellow maize and 18 in Chalqueño red maize when comparing agroecological and conventional systems. The direction of change was predominantly positive under agroecological management, with 16 metabolites increased in Zamorano yellow maize and 13 in Chalqueño red maize, while the remaining 4 and 5, respectively, exhibited the opposite trend. This asymmetric distribution indicates that each variety relies on distinct metabolic strategies shaped not only by the farming system but also by the local edaphoclimatic context where they were cultivated.

In Zamorano yellow cultivated in Contepec, characterized by more acidic soils and a colder climate due to its higher altitude. These conditions impose greater constraints on nutrient availability (especially phosphorus and nitrogen assimilation) and on enzymatic activity, which tends to slow down under lower temperatures. In this context, agroecological management enhanced the accumulation of organic acids (fumarate, succinate, citrate) and soluble sugars (glucose, maltose), together with stress-related metabolites such as GABA and glutamate. This pattern reflects a metabolic adjustment focused on reinforcing central carbon metabolism and osmotic regulation, ensuring energy supply and buffering capacity under acidic soils and cold stress. The relative decrease in amino acids under agroecological Zamorano yellow maize suggests that carbon flux was prioritized toward respiration and osmotic balance, rather than protein biosynthesis, a strategy consistent with adaptation to nutrient-limiting and temperature-challenging environments.

In contrast, Chalqueño red maize was cultivated in Zacapu, a region with richer organic soils and a more temperate climate, which provides a more favorable baseline for nutrient uptake and metabolic activity. Under agroecological management, Chalqueño red maize exhibited increased levels of amino acids, including branched-chain amino acids (valine, leucine, isoleucine), methionine, phenylalanine, and tryptophan, while several organic acids decreased. This indicates a redirection of carbon flux away from respiration toward secondary metabolism and amino acid-derived defenses. Phenylalanine and methionine serve as precursors for phenylpropanoids and methylated metabolites involved in antioxidant protection, lignification, and pathogen resistance. The favorable soil and climatic conditions in Zacapu likely facilitated this reallocation of resources, enabling the plant not only to sustain growth but also to enhance defense and nutraceutical compound biosynthesis, which is consistent with the significant yield increase observed for Chalqueño red maize under agroecological management.

Together, these results suggest that Zamorano yellow maize relies on strengthening energy metabolism and osmotic adjustment in response to acidic soils and cold climate, whereas Chalqueño red maize channels resources toward amino acid-derived defenses and phenolic metabolism in fertile, temperate soils. Both strategies are compatible with the concept of “metabolic priming” induced by agroecological systems, where improved soil microbial activity and organic inputs promote metabolite accumulation that enhances drought tolerance, immunity, and nutritional quality of the grain. This environment–management–genotype interaction underscores the importance of considering local soil and climate conditions when evaluating metabolic adaptations and productivity outcomes of traditional maize varieties under sustainable farming systems.

The yield values obtained indicate that agroecological management resulted in a marginal increase of 1.3% in Zamorano yellow maize, whereas red maize exhibited a substantial yield increase, corresponding to a relative increase of 250% compared to the conventional system. This percentage was calculated based on the relative difference between yields, using the following formula:

$$\text{Increase}\;\left(\%\right)=\frac{\left(Y_{\mathrm{agroecological}}-Y_{\mathrm{conventional}}\right)}{Y_{\mathrm{conventional}}}\times 100$$

Accordingly, average yield values of 3.65 t·ha^−1^ under conventional management and 12.8 t·ha^−1^ under agroecological management were considered. In absolute terms, this increase corresponded to an approximate difference of 9.15 t·ha^−1^ between both management systems for red maize. These results are consistent with previous reports on native maize cultivated under agroecological and conventional schemes. (De La Cruz et al., 2023). This behavior may be associated with the differences observed in key metabolic pathways related to central carbon and nitrogen metabolism, including alanine, aspartate and glutamate metabolism, pyruvate metabolism, and starch and sucrose metabolism. The differential activation of these pathways suggests a metabolic reorganization that may favor improved carbon assimilation, energy balance, and biomass accumulation. However, since nitrogen use efficiency was not directly evaluated in this study, this interpretation should be considered a metabolomic inference, supported by previous literature demonstrating that a more efficient balance between photosynthesis, respiration, and nutrient uptake can result in increased yields under agroecological systems. (Wilbois & Schmidt, 2019).

## 4 Conclusions

^1^H NMR-based metabolomic analysis revealed that agroecological and conventional management systems differentially modulate the metabolic composition of two native Mexican maize varieties, Zamorano yellow and Chalqueño red, in a variety-dependent manner.

In Zamorano yellow maize, agroecological management mainly affected pathways related to alanine, aspartate, and glutamate metabolism, as well as pyruvate metabolism and the tricarboxylic acid cycle, indicating a reorganization of primary and nitrogen-related metabolism. In contrast, Chalqueño red maize exhibited metabolic changes predominantly associated with starch and sucrose metabolism, galactose metabolism, and phenylalanine metabolism, suggesting a preferential modulation of carbohydrate metabolism and pathways linked to phenolic and structural compound biosynthesis.

Overall, agroecological management promoted the accumulation of key metabolites involved in central carbon and nitrogen metabolism, with positive implications for grain nutritional quality. These responses highlight the importance of management practices within their specific agroecological contexts in shaping maize metabolic profiles.

## Funding and acknowledgements

Funding This work was supported by Secretaría de Ciencia, Humanidades, Tecnología e Innovación (SECIHTI) PEE-2025-G78 granted to Y.C.-N and Secretaría de Ciencia, Humanidades, Tecnología e Innovación (SECIHTI) Infraestructura CONACyT 2019, 302670 granted Y.C.-N. The sponsors had no role in design and conduct of this study; collection, management, analysis, and interpretation of the data; or in preparation, review, or approval of the manuscript; or the decision to submit the manuscript for publication.

## CRediT authorship contribution statement

**Gustavo G. Medina-Mendoza:** Writing – review & editing, Writing – original draft, Visualization, Methodology, Investigation. **Elvia Becerra-Martínez:** Software, Methodology, Formal analysis, Data curation, Conceptualization. **Yair Cruz-Narváez:** Validation, Supervision, Project administration, Methodology, Funding acquisition, Conceptualization. **Gerardo Noriega-Altamirano:** Validation, Project administration, Methodology, Investigation, Funding acquisition. **José Javier Castro-Arellano:** Writing – review & editing, Visualization, Validation, Resources, Methodology, Conceptualization. **Oscar Camacho-Nieto:** Software, Resources. **Diego Hidalgo-Martínez:** Software, Resources.

## Declaration of competing interest

The authors declare that they have no known competing financial interests or personal relationship**s** that could have appeared to influence the work reported in this paper.

## Data Availability

The datasets used and/or analyzed during this study are available from the corresponding author upon reasonable request. Supplementary Tables and Figures are included as separate files.

## Appendix A Supplementary data

Supplementary material Image 1
