# Supplementary material for: “Effect of agroecological and conventional farming systems on the metabolomic profile of yellow and red maize assessed by 1H NMR”
Source: Food Chem X. 2026 Jan 7;33:103508. doi: 10.1016/j.fochx.2026.103508 (PMC12825075; doi:10.1016/j.fochx.2026.103508)
Supplement: Supplementary file 1 — Supplementary material [file mmc1.docx]

**SUPPLEMENTARY INFORMATION**

**“Effect of Agroecological and Conventional Farming Systems on the Metabolomic Profile of Yellow and Red Maize Assessed by ¹H NMR”**

Gustavo G. Medina-Mendoza^a^, Oscar Camacho-Nieto^b^, Diego Hidalgo-Martínez^c^, Gerardo Noriega-Altamirano^d^, José Javier Castro-Arellano^a^ , Yair Cruz-Narváez^a^*, Elvia Becerra-Martínez^e^*

*Corresponding author:

Yair Cruz Narváez, e-mail: [ycruzn@ipn.mx](mailto:ycruzn@ipn.mx)

Elvia Becerra Martínez, e-mail: [elmartinezb@ipn.mx](mailto:elmartinezb@ipn.mx)

**Table S1**. Metabolites detected by ^1^H-NMR in two different varieties of *Zea mays*.

| # | Metabolite | Chemical shifts (ppm), J (Hz), multiplicity |
| --- | --- | --- |
| ***Sugar*** | | |
| 1 | Fructose | 3.98 (m, 1H) CH-5, 4.02 (dd, *J*=12.7, 1.3) CH_2_-11 |
| 2 | Galactose | 4.63 (d, *J*=8.0, 1H) CH-2 |
| 3 | Glucose | 4.63 (d, *J*=7.9) CH-2, 5.22 (d, *J*=3.7) CH-2 |
| 4 | Glucose-1-phosphate | 5.25 (d, *J* = 4.5 Hz) CH-1 |
| 5 | Maltose | 5.21 (d, *J*=3.80) CH-11, 5.40 (d, *J*=3.89) CH-2 |
| 6 | Mannose | 5.21 (d J=2.2) CH-11 |
| 7 | Myo-inositol | 3.28 (t, *J*=9.4, 1H) CH-2 |
| 8 | Sucrose | 4.14 (d, *J*=3.1, 1H) CH-3, 5.42 (d, *J*=3.6, 1H) CH-7 |
| 9 | Xylose | 4.50 (d, *J* = 7.8 Hz) CH-1 |
| ***Amino acids*** | | |
| 10 | Alanine | 1.47 (d, *J*=7.2, 1H) CH_3_-6 |
| 11 | Arginine | 3.23 (t, *J*=6.93) CH_2_-6, 3.76 (t, *J*=6.11) CH-4 |
| 12 | Aspartate | 2.84 (dd, *J* = 17.5, 7.4 Hz), 2.94 (dd, *J* = 17.5, 3.7 Hz). |
| 13 | Asparagine | 2.85 (dd, *J*=16.9, 7.8, 1H) CH_2_-6, 2.95 (dd, *J*=16.9, 4.2, 1H) CH_2_-6 |
| 14 | GABA | 2.29 (t, *J*=7.4, 1H) CH_2_-4 |
| 15 | Glutamic acid | 2.3(s) CH, 3.8 (dd) CH |
| 16 | Glutamine | 2.10–2.15 (m, 2H) CH_2_-6, 2.40–2.46 (m, 1H) CH_2_-7 |
| 17 | Histidine | 8. 53 (s, 1H) CH-2 |
| 18 | Isoleucine | 0.93 (d, *J*=7.0, 3H) CH_3_-8, 1.00 (d, *J*=7.0, 1H) CH_3_-9 |
| 19 | Leucine | 0.95 (d, *J*=1.6, 1H) CH_3_-8, 0.96 (d, *J*=2.2, 1H) CH_3_-9 |
| 20 | Methionine | 2.12 (s) CH_3_-2, 2.64 (t, *J* = 7.6 Hz) CH_2_-3 |
| 21 | Phenylalanine | 7.31 (d, *J*=7.5) CH-3, 5, 7.35–7.37 (m) CH-4, 7.42 (t, *J*=7.5) CH-2, 6 |
| 22 | Proline | 2.01(m) |
| 23 | Threonine | 1.32 (d, *J*=6.6, 1H) CH_3_-8 |
| 24 | Tryptophan | 7.42 (d, *J*=8.0, 3H) CH-6, 7.72 (d, *J*=8.0, 1H) CH-7 |
| 25 | Tyrosine | 6.75 (d, *J*=7.18, 1H) CH-2, 6, 7.18 (d, *J*=7.18, 1H) CH-3, 5 |
| 26 | Valine | 0.98 (d, *J*=7.0, 1H) CH_3_-8, 1.03 (d, *J*=7.0, 1H) CH_3_-7 |
| 27 | 2-hidroxyisobutyrate | 1.36 (s) CH₃ (6H) |
| 28 | 2-hydroxybutyrate | 3.99 (dd, *J*=6.56, 4.52) CH-2 |
| ***Organic Acids*** | | |
| 29 | Acetic acid | 1.92 (s, 1H)) CH_3_ -4 |
| 30 | Citric acid | 2.71 (d, *J*=16.6, 1H) CH_2_-2- 5, 2.84 (d, *J*=15.6, 1H) CH_2_-2- 5 |
| 31 | Formic acid | 8.44 (s, 1H) CH-2 |
| 32 | Fumaric acid | 6.51 (s, 1H) CH-4, 5 |
| 33 | Lactic acid | 1.32 (d, *J* = 6.6 Hz) |
| 34 | Malic acid | 2.44 (dd, *J*=15.5, 9.9, 1H) CH_2_-5, 2.56 (dd, *J*=15.5, 3,2, 1H) CH_2_-5 |
| 35 | Pyruvic acid | 2.36 (s) |
| 36 | Succinic acid | 2.43 (s, 1H) 2.62 (s) CH_2_-2 |
| 37 | 3-O-Caffeoylquinic acid | 6.36 (H-80, d, *J*=15.9Hz) CH-15 |
| 38 | 4-O-Caffeoylquinic acid | 6.44 (H-80, d, *J*=15.9Hz) |
| 39 | 5-O-Caffeoylquinic acid | 6.41 (H-80, d, *J*=15.9Hz) |
| ***Alcohols*** | | |
| 40 | Ethanol | 1.17 (t, *J* = 7.1 Hz) |
| 41 | Propylene glycol | 1.13 (d, *J*=6.45) CH_3_-5 |
| ***Nucleosides*** | | |
| 42 | Adenine | 8.12 (s) CH-6 |
| 43 | Adenosine | 8.34 (s), 8.26 (s) |
| 44 | Cytidine | 7.88 (d, *J* = 7.2 Hz) |
| 45 | Guanosine | 7.99 (s) |
| 46 | Uridine | 7.86 (d, *J* = 8.1 Hz) |
| ***Other metabolites*** | | |
| 47 | Choline | 3.19 (s, 1H) CH_3_-5, 6, 7 |
| 48 | Sn-glycero-3-phosphocholine | 3.2 (s) CH_3_-6, 13, 14, 4.3 (m) CH_2_-3 |
| 49 | Trigonelline | 8.85 (d, *J* = 6.1 Hz) CH-3, 8.90 (d, *J* = 8.0 Hz) CH-5, 9.17 (s) CH |

s = singlet, d = doublet, t = triplet, dd = doublet of doublets, m = multiplet.

The numbering of the molecules was taken from the HMDB database (<http://www.hmdb.ca>).

**Table S2.** Metabolites with the highest significance identified by VIP scores in Zamorano yellow maize

| # | Metabolite | VIP score |
| --- | --- | --- |
| 1 | Acetic acid | 1.4025 |
| 2 | Methionine | 1.3618 |
| 3 | Aspartate | 1.3567 |
| 4 | Maltose | 1.3472 |
| 5 | Arginine | 1.3411 |
| 6 | Fumaric acid | 1.3368 |
| 7 | 4-O-Caffeoylquinic acid | 1.2898 |
| 8 | Glucose-1-phosphate | 1.2887 |
| 9 | Ethanol | 1.2451 |
| 10 | Sn-glycero-3-phosphocholine | 1.2391 |
| 11 | Propylene glycol | 1.1288 |
| 12 | Succinic acid | 1.0998 |
| 13 | Citric acid | 1.0668 |
| 14 | Adenosine | 1.0499 |
| 15 | Adenine | 1.0415 |
| 16 | Tyrosine | 1.0361 |
| 17 | Proline | 1.0348 |
| 18 | 3-O-Caffeoylquinic acid | 1.0277 |
| 19 | 2-hydroxybutyrate | 1.0191 |
| 20 | GABA | 1.0112 |

**Table S3.** Metabolites with the highest significance identified by VIP scores in Chalqueño red maize

| # | Metabolite | VIP score |
| --- | --- | --- |
| 1 | Tryptophan | 1.4814 |
| 2 | 4-O-Caffeoylquinic acid | 1.4713 |
| 3 | Methionine | 1.3316 |
| 4 | Acetic acid | 1.3217 |
| 5 | Glucose | 1.3013 |
| 6 | Adenine | 1.288 |
| 7 | Propylene glycol | 1.2792 |
| 8 | Leucine | 1.2553 |
| 9 | Isoleucine | 1.2512 |
| 10 | Phenylalanine | 1.2374 |
| 11 | Glutamic acid | 1.2067 |
| 12 | Valine | 1.2044 |
| 13 | Guanosine | 1.1796 |
| 14 | Threonine | 1.1513 |
| 15 | Choline | 1.1495 |
| 16 | Sucrose | 1.1261 |
| 17 | Alanine | 1.0634 |
| 18 | Adenosine | 1.0587 |

**Table S4**. Results of pathway analysis of Zamorano yellow maize (MZ) agroecological and conventionally.

| **#** | **Pathway** | **Raw p** | **-log (P)** | **Holm adjust** | **FDR** | **Impact** | **Importance** | | **Matched metabolites** |
| --- | --- | --- | --- | --- | --- | --- | --- | --- | --- |
| 1 | Glyoxylate and dicarboxylate metabolism | 3.00E-07 | 6.5233 | 1.74E-05 | 1.74E-05 | 0.1858 | Malic acid  Formic acid  Citric acid  Glutamic acid | 0.0640  0.0013  0.0870  0.0332 | 4 |
| 2 | Alanine, aspartate and glutamate metabolism | 2.53E-06 | 5.5963 | 1.44E-04 | 4.93E-05 | 0.77698 | Aspartic acid  L-glutamine  Fumaric acid  Glutamic acid  GABA | 0.1259  0.1942  0.0036  0.3237  0.1295 | 5 |
| 3 | Pyruvate metabolism | 2.55E-06 | 5.5931 | 1.44E-04 | 4.93E-05 | 0.38783 | Acetic acid  Malic acid | 0.0957  0.1435 | 2 |
| 4 | Arginine biosynthesis | 1.66E-05 | 4.7804 | 9.12E-04 | 2.40E-04 | 0.25977 | Glutamic acid  L-arginine  L-glutamine | 0.0997  0.1316  0.0282 | 3 |
| 5 | Butanoate metabolism | 2.46E-05 | 4.6098 | 0.0013263 | 2.85E-04 | 0.13636 | GABA | 0.1363 | 1 |
| 6 | Glycolysis or Gluconeogenesis | 6.79E-05 | 4.1679 | 0.0035329 | 5.36E-04 | 0.11812 | Acetic acid  Pyruvate | 0.0014  0.1166 | 2 |
| 7 | Citrate cycle (TCA cycle) | 7.39E-05 | 4.1312 | 0.0037706 | 5.36E-04 | 0.21839 | Malic acid  Succinic acid  Fumaric acid  Citric acid | 0.0330  0.0401  0.0295  0.1157 | 4 |
| 8 | Sulfur metabolism | 9.39E-05 | 4.0274 | 0.0046945 | 6.05E-04 | 0.07407 | L-tyrosine  Fumaric acid | 0.1675  0.0702 | 2 |
| 9 | Tyrosine metabolism | 1.75E-04 | 3.757 | 0.0085741 | 0.0010149 | 0.23784 | L-arginine  L-proline | 0.0917  0.0336 | 2 |
| 10 | Arginine and proline metabolism | 3.82E-04 | 3.4174 | 0.018358 | 0.0020166 | 0.12538 | D-glucose1-phospate  D-fructose  Sucrose  Maltose  D-glucose | 0.0904  0.0158  0.0888  0.0999  0.3003 | 5 |
| 11 | Starch and sucrose metabolism | 9.98E-04 | 3.0008 | 0.045913 | 0.0044531 | 0.59548 | D-glucose 1-phospate  D-galactose | 0.0181  0.3311 | 2 |

**Table S5**. Results of pathway analysis of Chalqueño red maize organically and conventionally

| # | Pathway | Raw p | -log (P) | Holm adjust | FDR | Impact | Importance | | **Matched metabolites** |
| --- | --- | --- | --- | --- | --- | --- | --- | --- | --- |
| 1 | Valine, leucine and isoleucine biosynthesis | 8.32E-06 | 5.0801 | 4.74E-04 | 2.41E-04 | 0.10727 | Pyruvate | 0.1072 | 1 |
| 2 | Glyoxylate and dicarboxylate metabolism | 1.51E-04 | 3.8196 | 0.0080292 | 0.0014645 | 0.1858 | Malate  Citrate  Formate  L-glutamate | 0.0640  0.0870  0.0013  0.0332 | 4 |
| 3 | Amino sugar and nucleotide sugar metabolism | 1.92E-04 | 3.7176 | 0.009964 | 0.0015877 | 0.10224 | D-glucose 1-phosphate | 0.1024 | 1 |
| 4 | Glycine, serine and threonine metabolism | 2.92E-04 | 3.5348 | 0.014885 | 0.002116 | 0.123 | L-threonine | 0.1230 | 1 |
| 5 | Starch and sucrose metabolism | 3.34E-04 | 3.4763 | 0.016697 | 0.002152 | 0.59548 | D-glucose 1-phosphate  D-fructose  Sucrose  Maltose  D-glucose | 0.0904  0.0158  0.0888  0.0999  0.3003 | 5 |
| 6 | Fructose and mannose metabolism | 4.22E-04 | 3.375 | 0.020661 | 0.0022405 | 0.10151 | D-fructose  D-manose | 0.0669  0.0345 | 2 |
| 7 | Phenylalanine metabolism | 0.001907 | 2.7197 | 0.085967 | 0.0073737 | 0.42308 | L-phenylalanine | 0.4230 | 1 |
| 8 | Galactose metabolism | 0.0023106 | 2.6363 | 0.099358 | 0.0083761 | 0.34927 | D-glucose 1-phosphate  D-galatose | 0.0181  0.3311 | 2 |
| 9 | Tryptophan metabolism | 0.0030826 | 2.5111 | 0.12947 | 0.0099328 | 0.1938 | L-tryptophan | 0.1938 | 1 |
| 10 | Butanoate metabolism | 0.0033703 | 2.4723 | 0.13481 | 0.010288 | 0.13636 | GABA | 0.1363 | 1 |


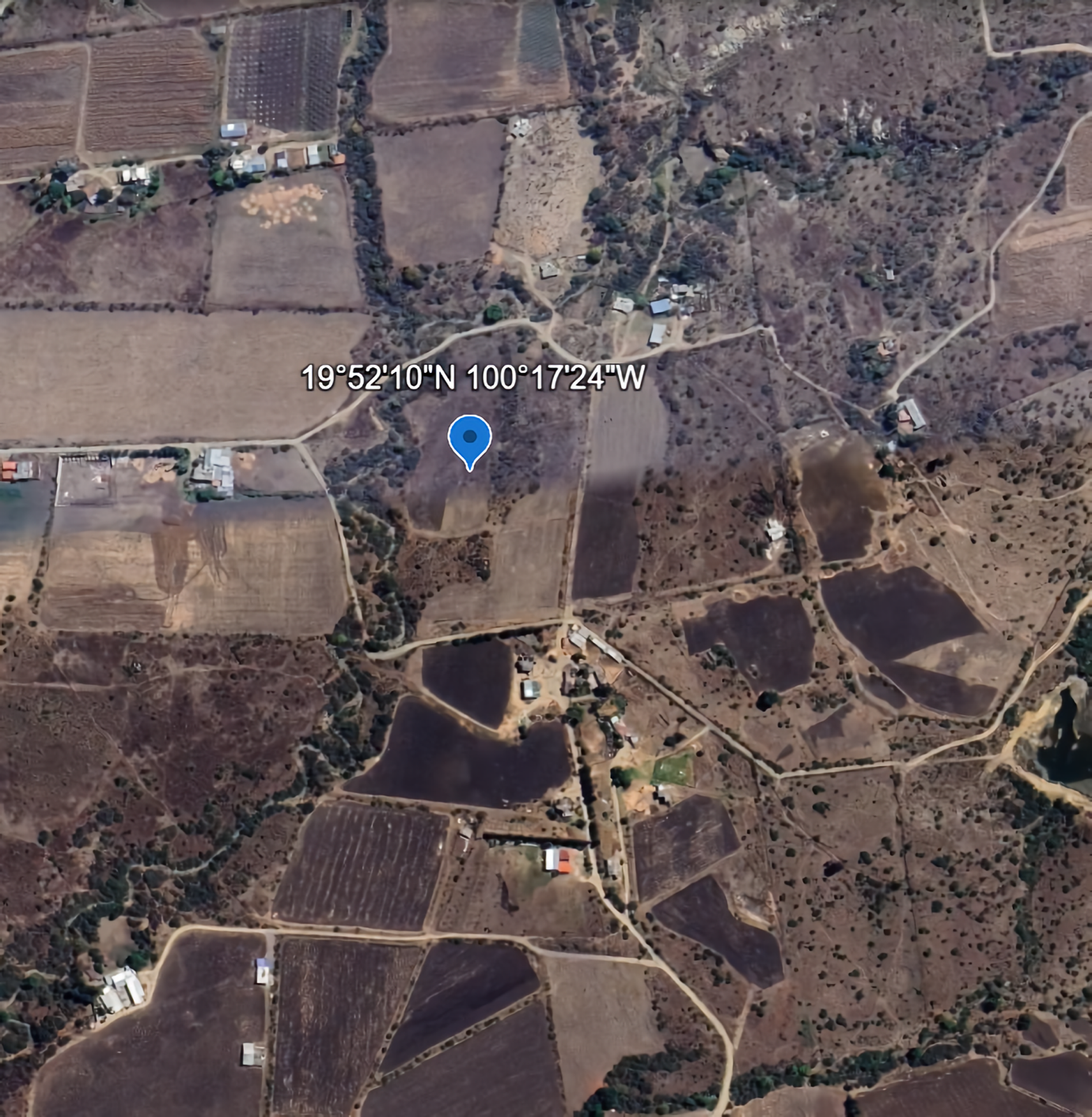


**Fig. S1.** Satellite image of the Municipality of Contepec, Michoacán, Mèxico. (Google, n.d.).


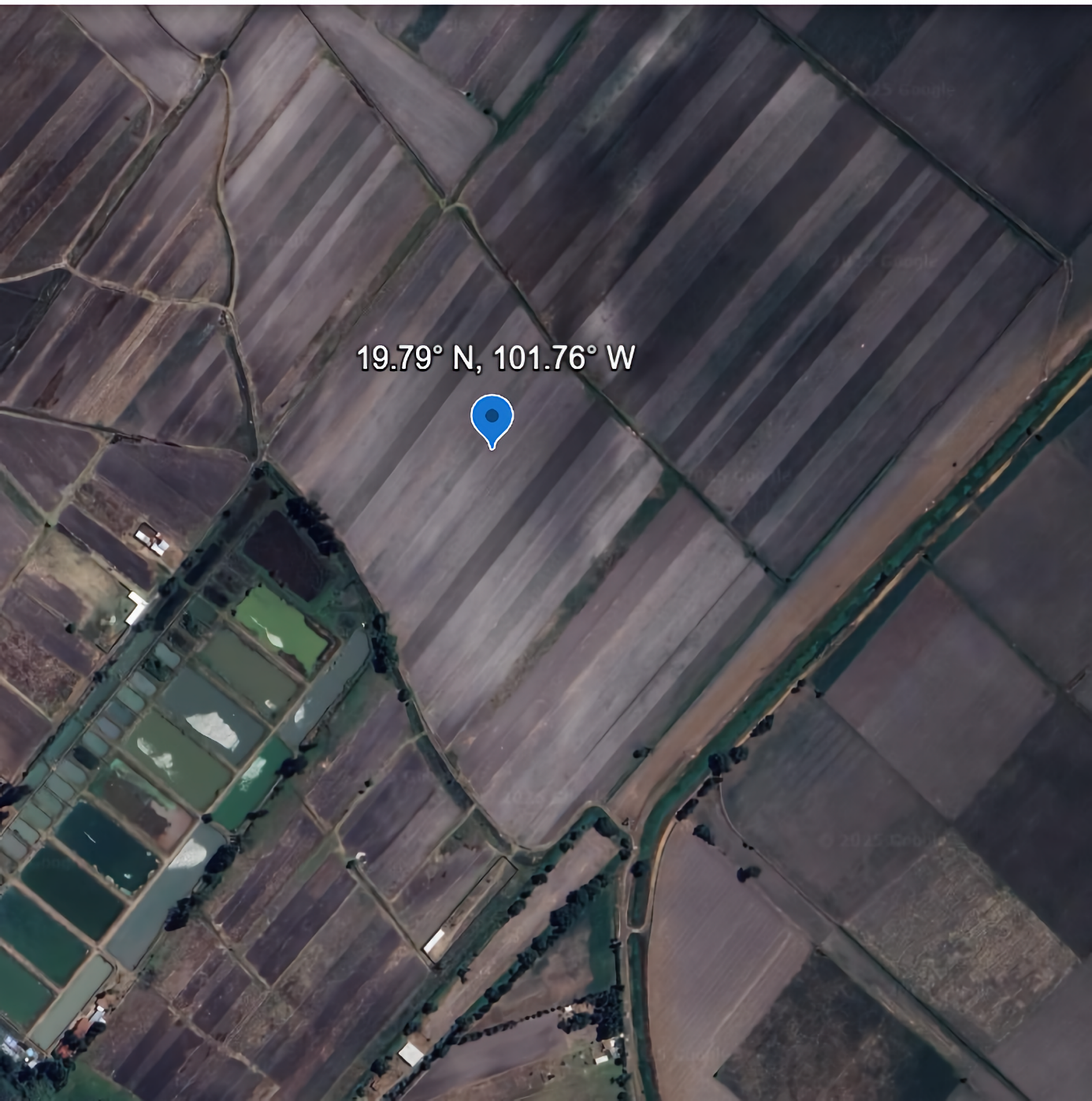


**Fig. S2.** Satellite image of the Municipality of Zacapu, Michoacán, Mèxico. (Google, n.d.)


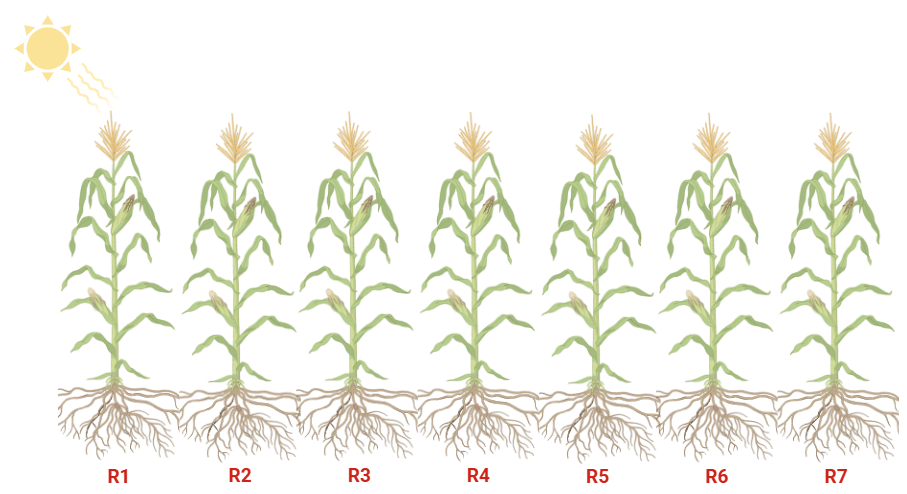


**Fig. S3.** Collection of seven representative specimens per row and variety


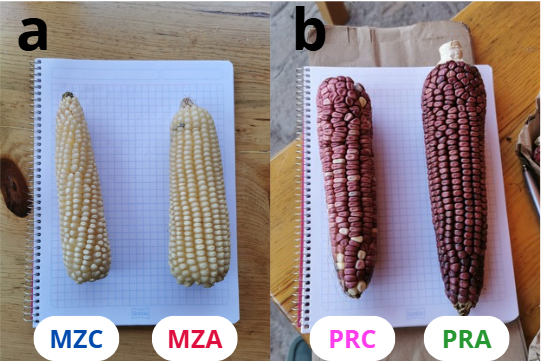


**Fig. S4.** Arrangement of collected ears: (a) conventional Zamorano yellow maize (MZC), agroecological Zamorano yellow maize (MZA), (b) conventional Chalqueño red maize (PRC), and agroecological Chalqueño red maize (PRA).


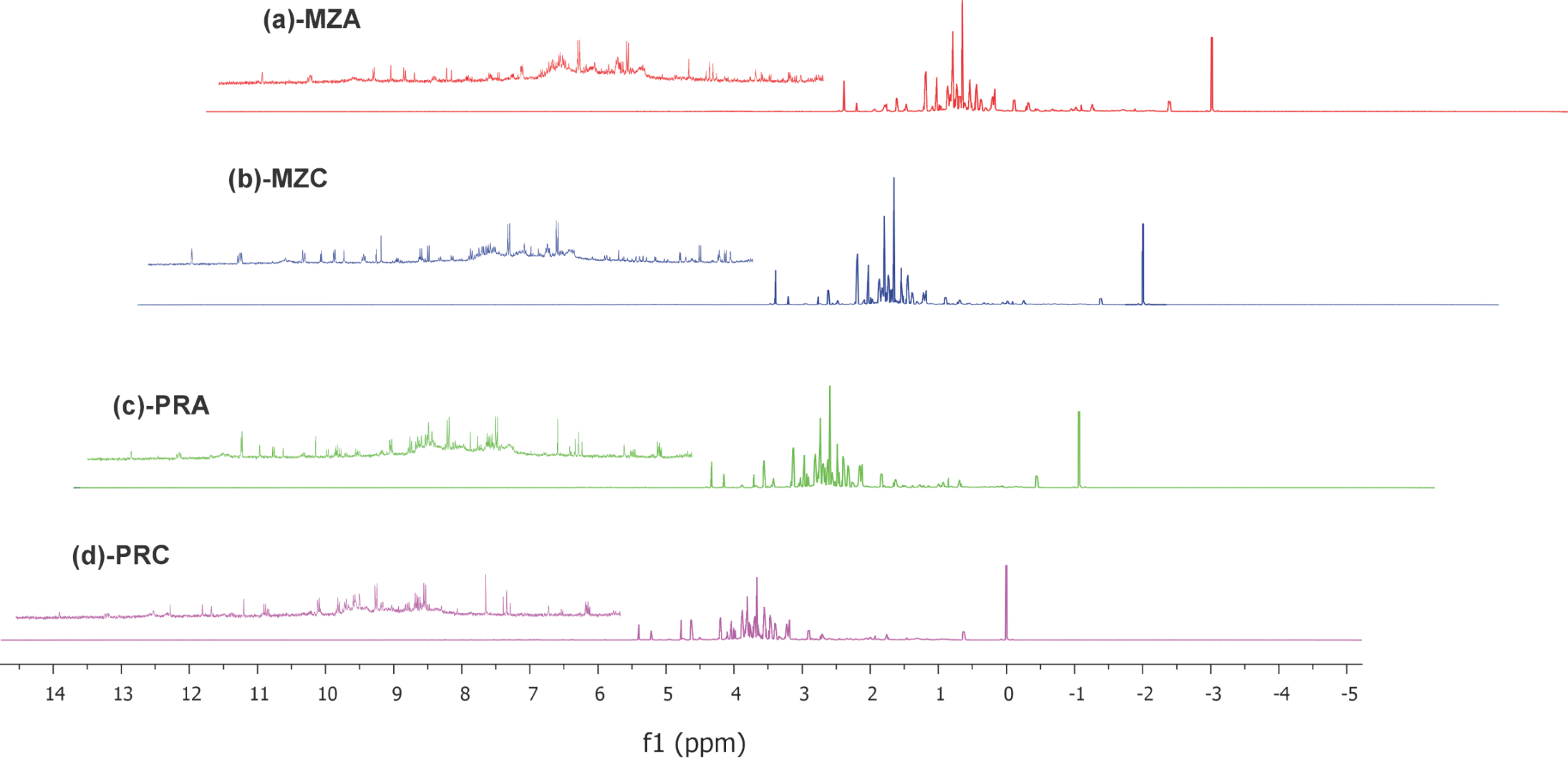


**Fig. S5.** ¹H NMR spectra of aqueous extracts from two maize varieties under different cultivation systems: (a) MZA – agroecological Zamorano yellow maize, (b) MZC – conventional Zamorano yellow maize, (c) PRA – agroecological Chalqueño red maize, and (d) PRC – conventional Chalqueño red maize. Spectra obtained at 750 MHz were scaled according to TSP used as the internal standard.


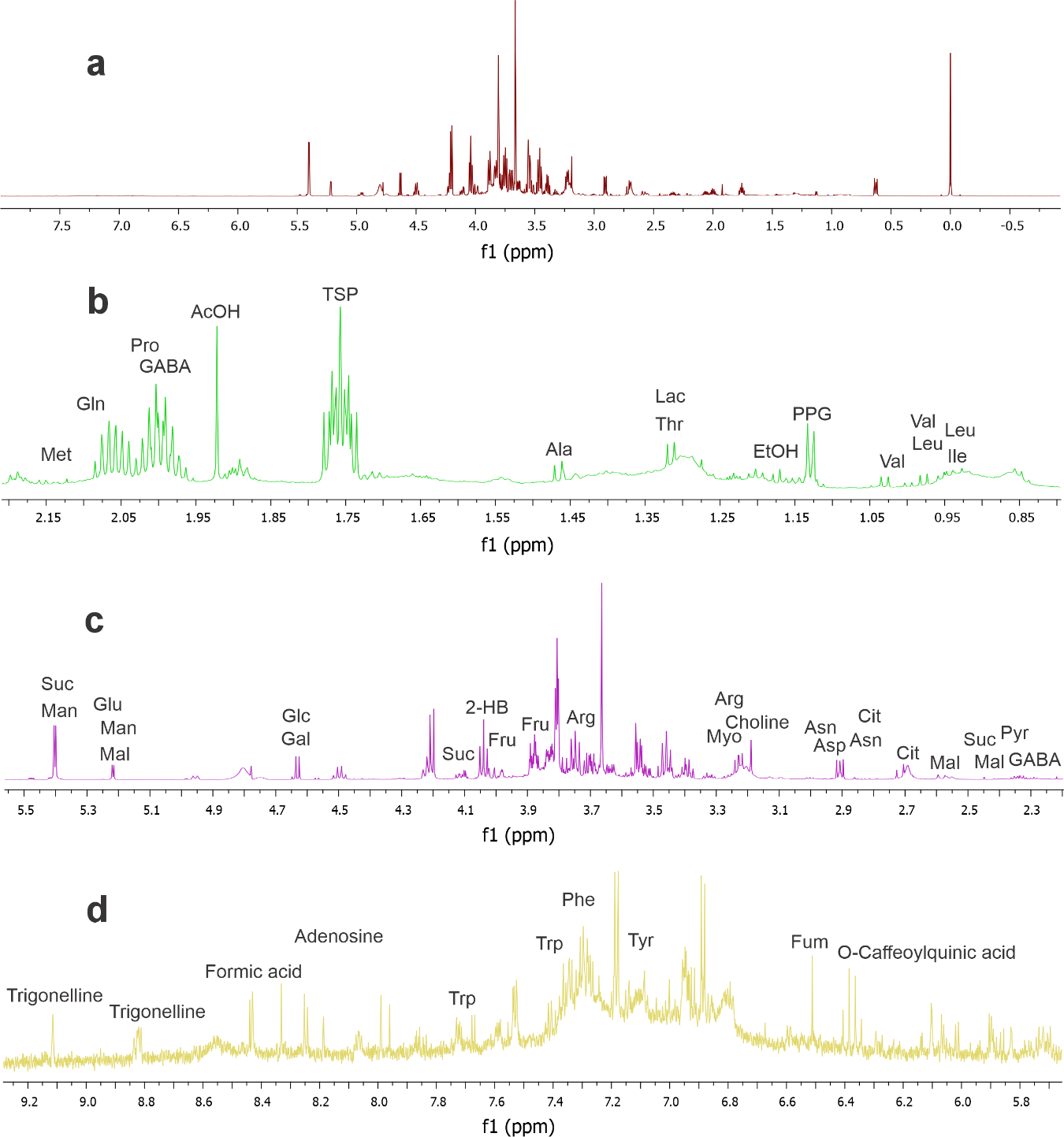


**Fig. S6**. Characteristic ¹H NMR spectrum obtained at 750 MHz from representative aqueous maize extracts. Signal assignments were based on two-dimensional (2D) NMR experiments and literature data.


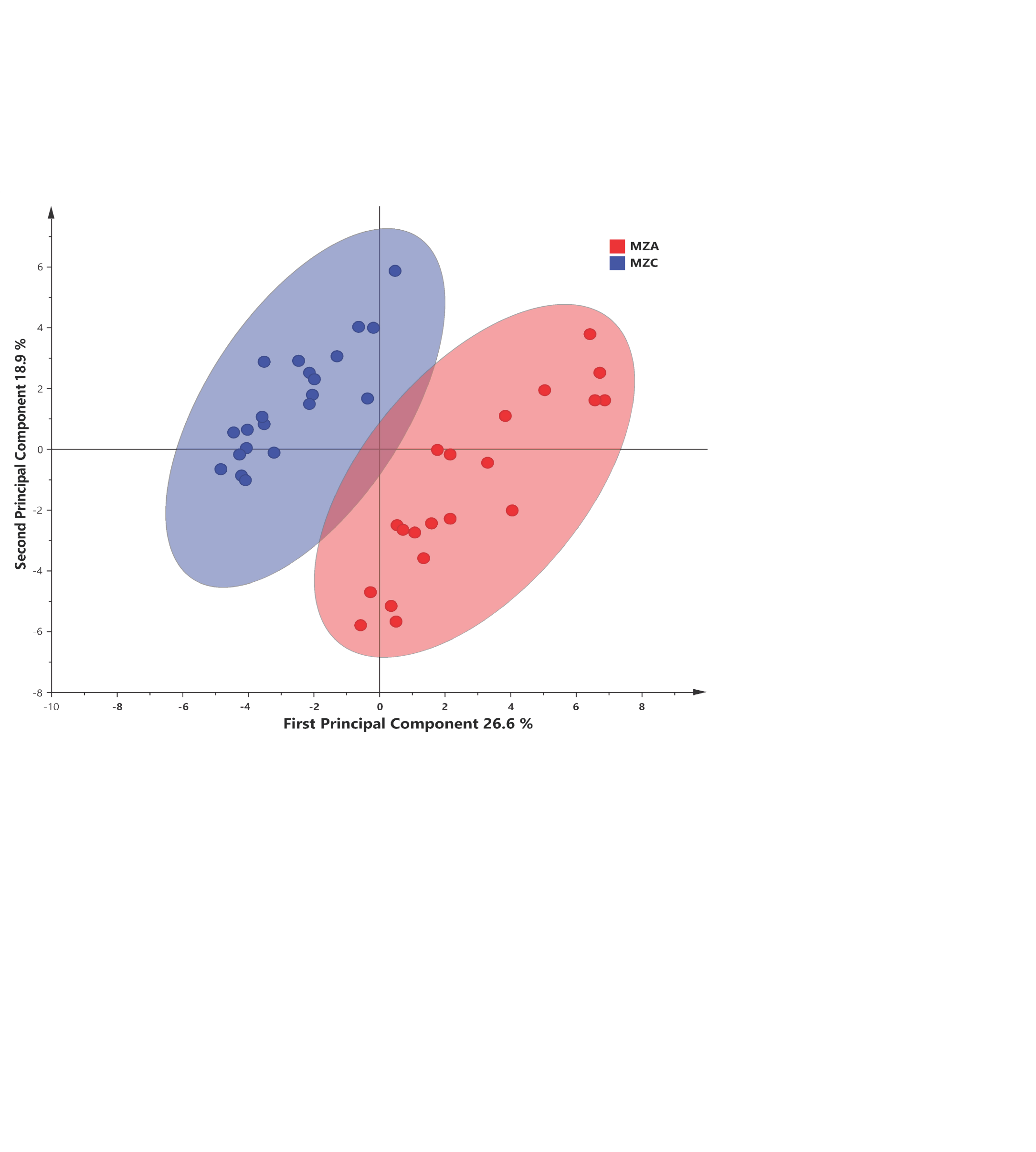


**Fig. S7.** PCA score plot generated from the ^1H NMR spectra (750 MHz) of Zamorano yellow maize (MZ) samples cultivated under agroecological (MZA, red) and conventional (MZC, blue) management systems. Each point represents an independent biological replicate. The ellipses correspond to the 95% confidence interval for each group, illustrating within-group variability and the separation between management systems.


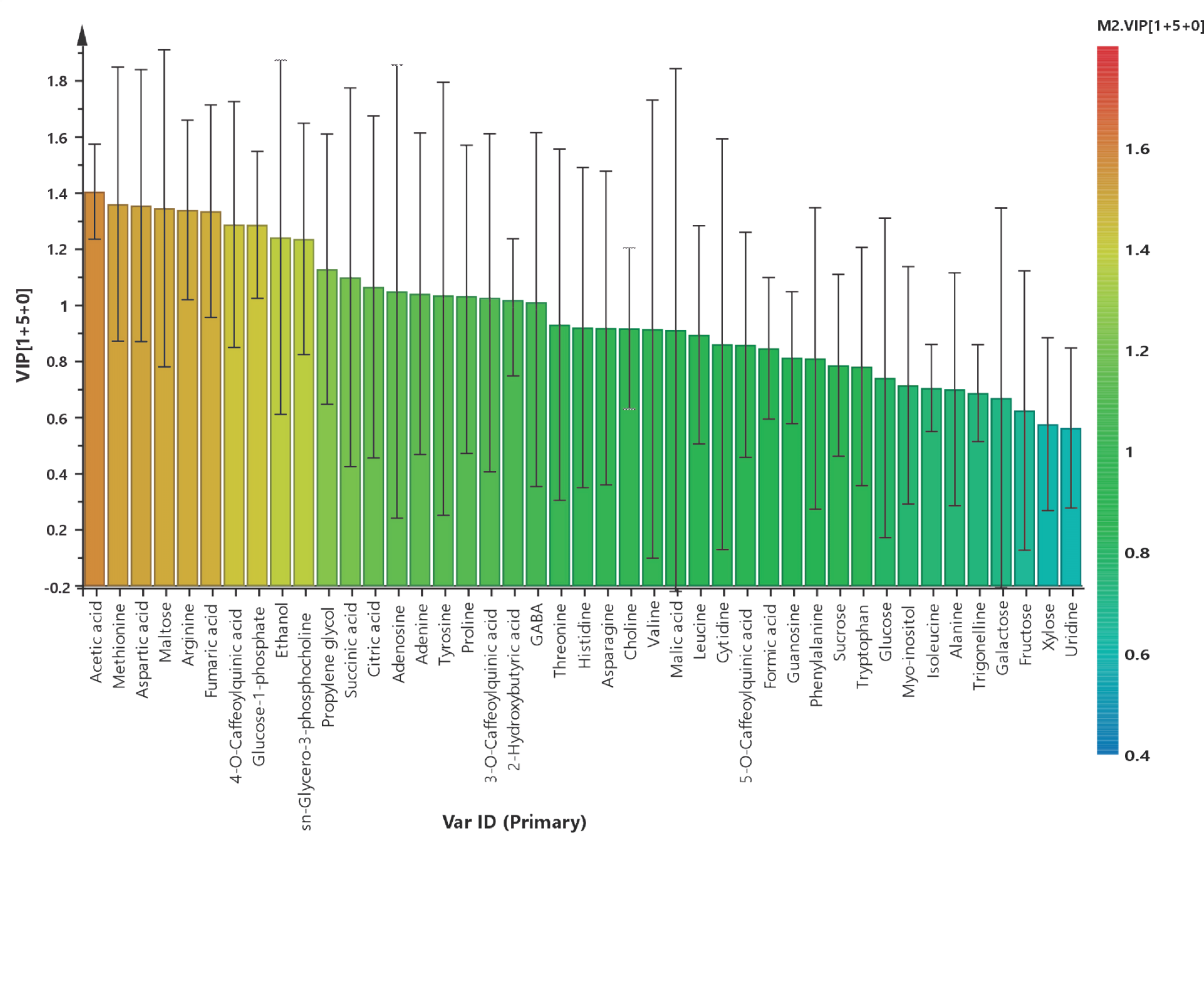


**Fig. S8**. VIP scores of OPLS-DA of maize Zamorano.


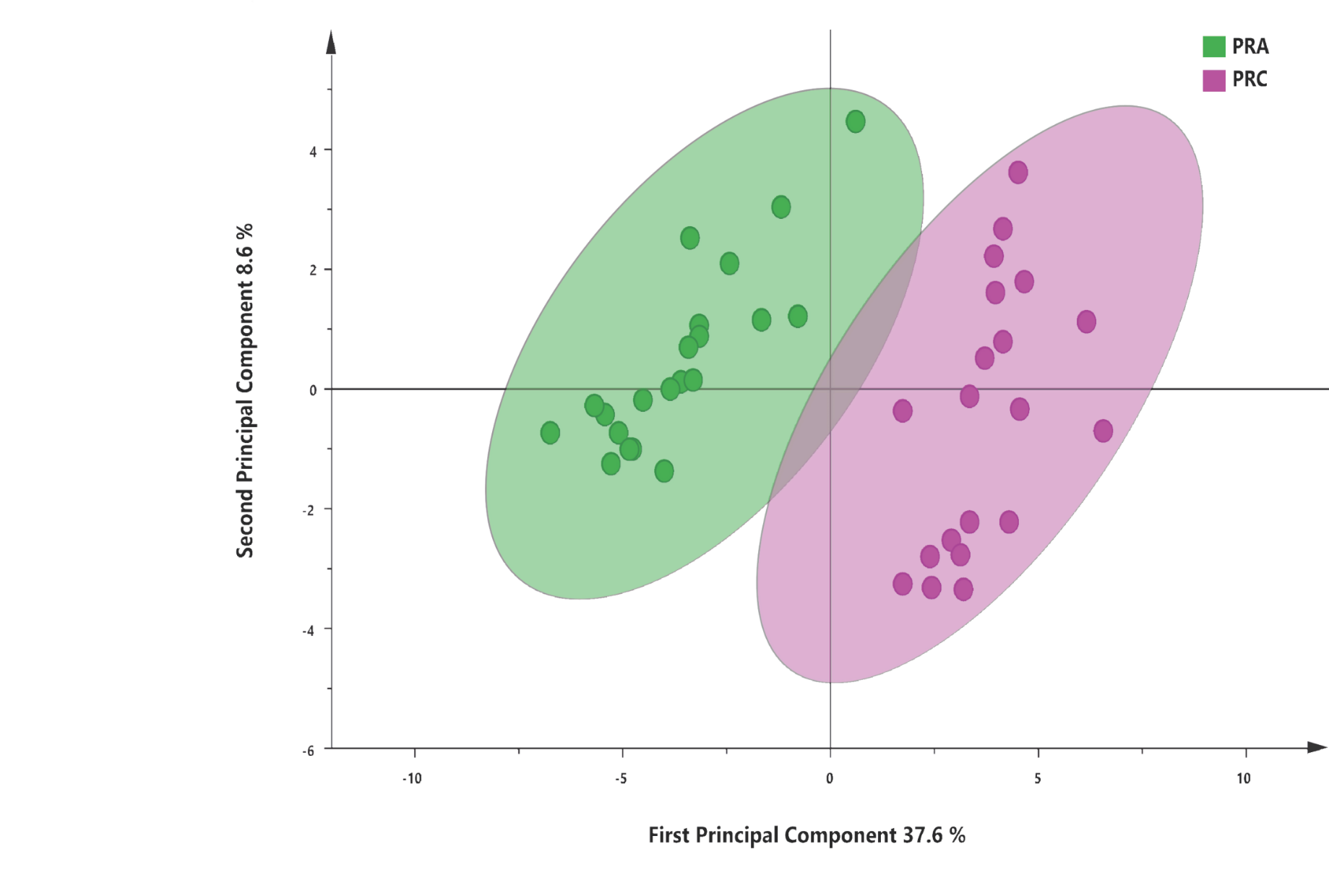


**Fig. S9.** PCA score plot generated from the ^1^H NMR spectra (750 MHz) of Chalqueño red maize (PR) samples cultivated under agroecological (PRA, green) and conventional (PRC, pink) management systems. Each point represents an independent biological replicate. The ellipses correspond to the 95% confidence interval for each group, illustrating within-group variability and the separation between management systems.


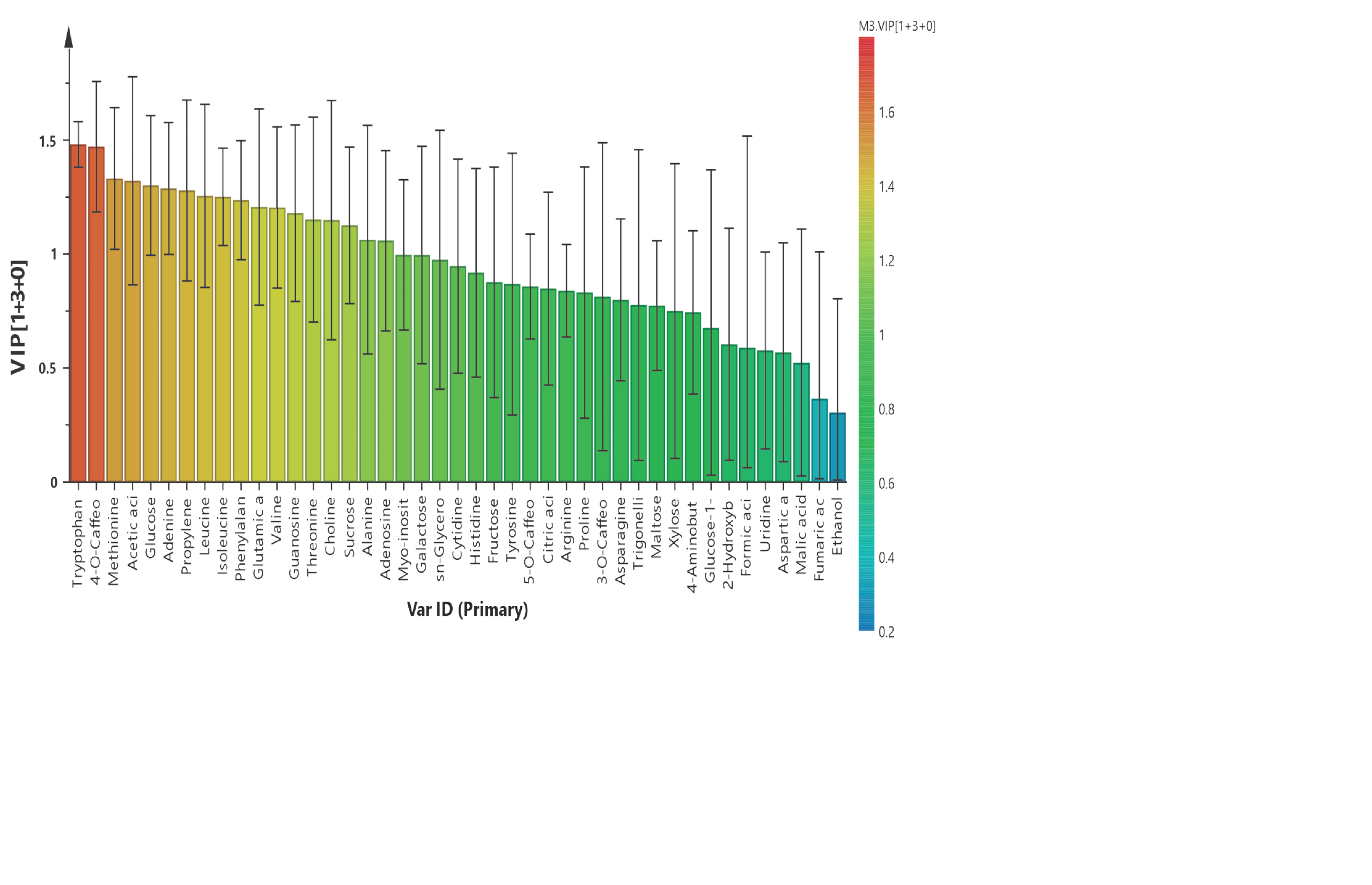


**Fig. S10.** VIP scores of OPLS-DA of Chalqueño red maize.


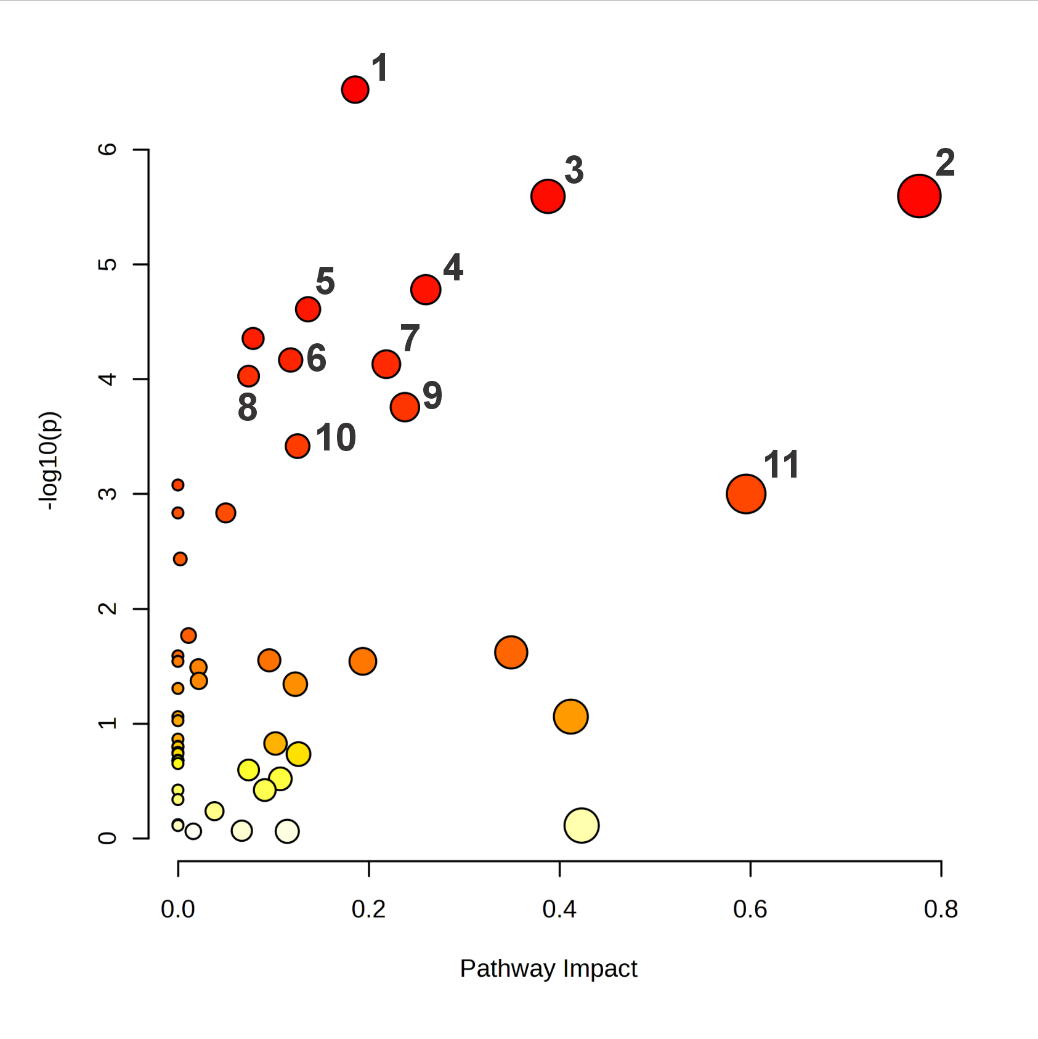


**Fig. S11.** Metabolic pathway alterations in Zamorano yellow maize (MZ) cultivated under agroecological and conventional systems, analyzed using MetaboAnalyst 6.0. Differences were considered statistically significant at p < 0.05 with a topological impact threshold > 0.1. The main affected pathways are: (1) Glyoxylate and dicarboxylate metabolism, (2) Alanine, aspartate, and glutamate metabolism, (3) Pyruvate metabolism, (4) Arginine biosynthesis, (5) Butanoate metabolism, (6) Glycolysis or gluconeogenesis, (7) Citric acid cycle (TCA cycle), (8) Sulfur metabolism, (9) Tyrosine metabolism, (10) Arginine and proline metabolism, and (11) Starch and sucrose metabolism.


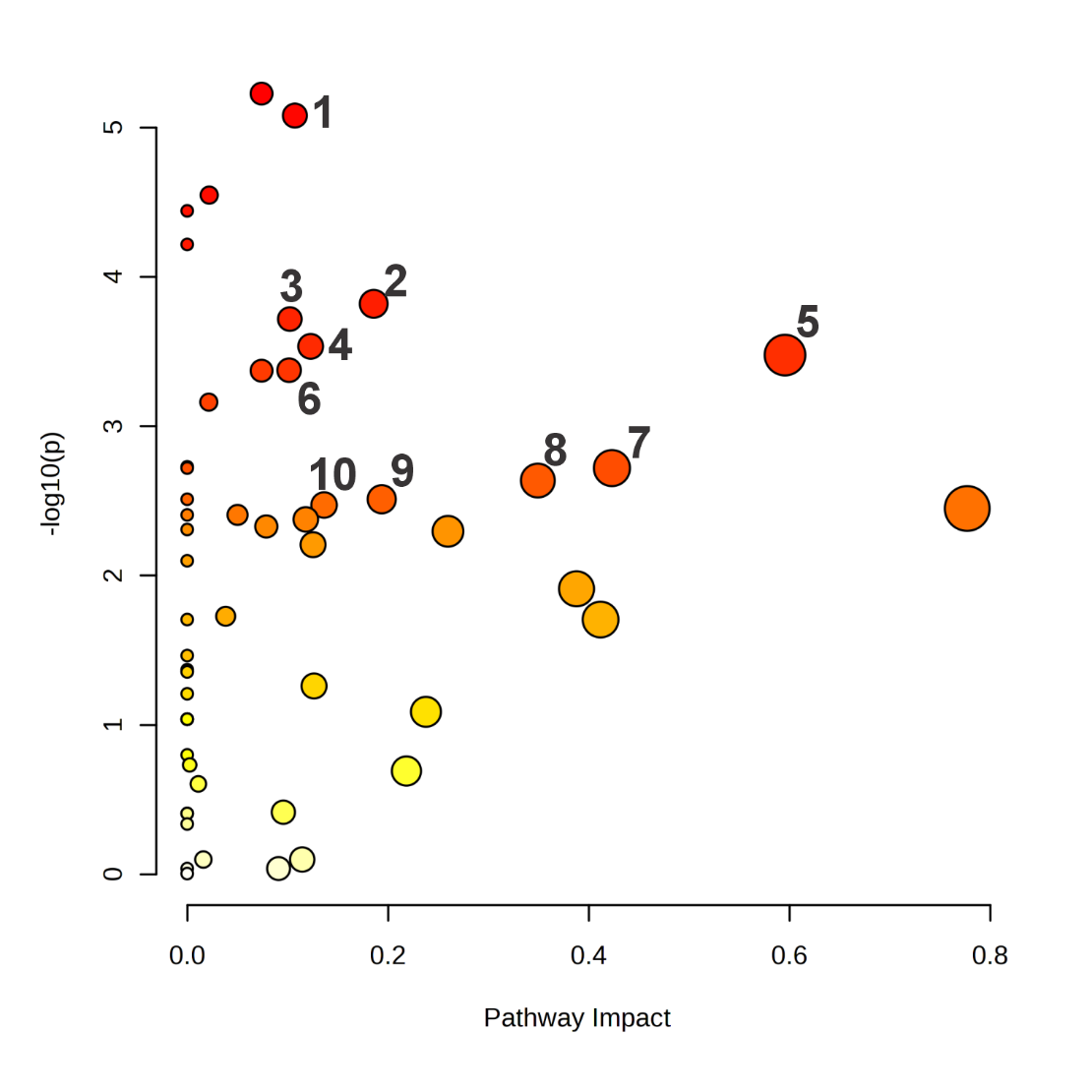


**Fig. S12.** Metabolic pathway alterations in Chalqueño red maize (PR) cultivated under agroecological and conventional systems, analyzed using MetaboAnalyst 6.0. Differences were considered statistically significant at p < 0.05 with a topological impact threshold > 0.1. The main affected pathways are: (1) Valine, leucine, and isoleucine biosynthesis, (2) Glyoxylate and dicarboxylate metabolism, (3) Amino sugar and nucleotide sugar metabolism, (4) Glycine, serine, and threonine metabolism, (5) Starch and sucrose metabolism, (6) Fructose and mannose metabolism, (7) Phenylalanine metabolism, (8) Galactose metabolism, (9) Tryptophan metabolism, and (10) Butanoate metabolism.
